# Real-World Electronic Medical Records Data Identify Risk Factors for Myelofibrosis and Can Be Used to Validate Established Prognostic Scores

**DOI:** 10.3390/cancers16071416

**Published:** 2024-04-05

**Authors:** Max Kappenstein, Nikolas von Bubnoff

**Affiliations:** Medical Center, Department of Hematology and Oncology, University Hospital Schleswig-Holstein, Campus Lübeck, Ratzeburger Allee 160, 23538 Lübeck, Germany

**Keywords:** hematology, myelofibrosis, malignancies, risk factors

## Abstract

**Simple Summary:**

Myelofibrosis is a rare bone marrow disorder, leading to an increasing failure to generate healthy blood cells. Defining clinical prognosis scores for rare diseases is difficult, as sufficient numbers of patients for score validation are difficult to obtain. The current study investigates the utility of the TriNetX database, containing electronic medical records for over 140 million patients, to identify risk factors and establish clinical scores. TriNetX includes more than 64,000 myelofibrosis patients, and the present study explores factors influencing survival and common complications. Age over 65, anemia, an increased number of leukocytes, a low platelet count and an increased number of monocytes are associated with increased risks, while high numbers of eosinophiles and basophiles show positive associations. We demonstrate that the TriNetX database offers insights to refine predictive models, crucial for tailoring treatments to individual patient risks in the complex landscape of rare diseases like myelofibrosis.

**Abstract:**

Myelofibrosis (MF) is a myeloproliferative neoplasia arising de novo as primary myelofibrosis (PMF) or secondary to polycythemia vera or essential thrombocythemia. Patients experience a high symptom burden and a marked reduction in life expectancy. Despite progress in molecular understanding and treatment, the clinical and prognostic heterogeneity of MF complicates treatment decisions. The International Prognostic Scoring System (IPSS) integrates clinical factors for risk stratification in MF. This study leverages the TriNetX database with more than 64,000 MF patients to assess the impact of accessible parameters on survival and complicating events, including AML transformation, cachexia, increased systemic inflammation, thrombosis and hemorrhage. Age over 65 years correlated with increased risks of death, AML transformation, thrombosis and hemorrhage. Anemia (Hb < 10 g/dL), leukocytosis (>25 × 10^3^/µL) and thrombocytopenia (<150 × 10^3^/µL) reduced survival and increased risks across all assessed events. Monocytosis is associated with decreased survival, whereas eosinophilia and basophilia were linked to improved survival. Further, as proof of concept for the applicability of TriNetX for clinical scores, we devised a simplified IPSS, and confirmed its value in predicting outcomes. This comprehensive study underscores the importance of age, anemia, leukocytosis and thrombocytopenia in predicting disease trajectory and contributes to refining prognostic models, addressing the challenges posed by the disease’s heterogeneity.

## 1. Introduction

Myelofibrosis (MF) is a myeloproliferative neoplasm (MPN) consisting of two distinct entities. While primary Myelofibrosis (PMF) arises de novo and without known precipitating condition, secondary Myelofibrosis occurs as stage of disease progression in individuals with polycythemia vera (PV) and essential thrombocythemia (ET). MF is characterized by a progressing bone marrow fibrosis which leads to a subsequent disturbance of physiological hematopoiesis [1]. Its characteristic clinical presentation includes anemia, hepatosplenomegaly, cachexia, bone pain and constitutional symptoms. Frequent causes of death include transformation to acute myeloid leukemia (AML), cachexia, infections, thrombosis and hemorrhage [2]. Dysregulated inflammatory cytokine production and increased JAK-STAT signaling play a pivotal role in pathogenesis and clinical presentation of the disease. The JAK kinase inhibitors ruxolitinib and fedratinib have been demonstrated to decrease spleen size and improve symptom burden, and were therefore approved for the treatment of MF [3,4,5].

Despite advancements in understanding its molecular basis, the heterogeneity of clinical courses in MF poses challenges in predicting disease outcomes and optimizing treatment strategies. Treatment is based on the patient’s risk profile, symptoms, age and comorbidities. Patients with a low risk of progression might undergo watch-and-wait or JAK inhibitor treatment in case of symptoms, while patients with a high risk of progression should undergo allogeneic stem cell transplantation [6,7]. Accordingly, determining the patient’s risk profile is crucial for treatment stratification.

A pivotal contribution towards enhancing prognostication in PMF has been the development of the International Prognostic Scoring System (IPSS) by Cervantes et al. [8] which integrates five clinical factors (age > 65 years, hemoglobin < 10 g/dL, leukocyte count > 25 × 10^3^/µL, circulating blasts ≥ 1%, and constitutional symptoms) to stratify patients into distinct risk categories. This classification has proven valuable for predicting survival outcomes at the time of diagnosis and thus guides treatment decisions. In order to assess survival at any time during the clinical course, it was refined to accommodate risk-stratifying factors as dynamic IPSS (DIPSS) [9], extended to DIPSS plus by the inclusion of cytogenetic factors [10]. These scoring systems established for PMF are also used for secondary MF despite known discrepancies in assessing survival [11]. In order to address these limitations, the Myelofibrosis Secondary to Polycythemia Vera and Essential Thrombocythemia-Prognostic Model (MYSEC-PM) was devised, which includes hemoglobin < 11 g/dL, circulating blasts > 2%, CALR unmutated, thrombocyte count < 150 × 10^3^/µL, any year of age and the presence of constitutional symptoms as risk factors [12]. The mutation-enhanced IPSS (MIPSS70) incorporates high-molecular-risk mutations in patients below the age of 70 years [13].

However, with an incidence of 0.5–1.5 per 100,000 inhabitants per year [14,15,16], classical MPN are rare diseases, making it difficult to assess the impact of prognostic factors through a sufficiently large cohort of patients and to determine further clinical factors to assess patient risk. In this study, we leveraged the TriNetX database to determine the impact of criteria of the clinical MF scores accessible through the platform, which are age, hemoglobin, leukocyte count, platelet count as well as further laboratory parameters at the time of diagnosis on MF outcome and their impact on complications.

## 2. Methods

### 2.1. Data Source

The present study used the TriNetX federate research network which offers a collection of electronic medical records (EMRs) from 115 international healthcare organizations (HCOs) in March 2024, including academic medical institutions, specialty physician services and community hospitals. These EMRs include information on diagnoses, procedures, medications, laboratory values and partially genomic data [17,18,19,20] of 143 million patients as of March 2024.

### 2.2. Study Design

The present study is a retrospective study which identified patients at their first diagnosis of osteomyelofibrosis (D47.4) and compared their outcomes using TriNetX EMRs. The study included MF patients irrespective of age at diagnosis with at least one documented visit after the follow-up period or with documented death. The study excluded patients that met the index event less than 5 or more than 20 years ago.

For each parameter assessed, the patients were classified in two cohorts by criteria indicated in Table 1. The thresholds for age, hemoglobin, leukocytes and platelets were chosen as in the study establishing the original IPSS [8]. In addition, we assessed the impact of elevated monocyte, basophil and eosinophil counts due to their association with outcome in CML and mastocytosis [21,22,23,24]. The definition of basophilia, eosinophilia and monocytosis are in line with thresholds established in the literature [25,26,27]. The patient cohort characteristics are shown in Supplementary Table S1.

In order to avoid confounding, the cohorts were matched through propensity score matching provided by TriNetX, which employs user-defined covariates to create input matrices and then conducts logistic regression analysis to generate propensity scores for individual subjects. These propensity scores are used to perform 1:1 matching using greedy nearest neighbor algorithms, with a caliper width of 0.1 pooled standard deviations. To eliminate bias resulting from the nearest neighbor algorithms, TriNetX randomizes the order of rows. This method has been validated previously [28]. Propensity score matching was performed regarding sex and race and, for the assessments of independent parameters, for the other assessed variables to avoid confounding.

Diagnosis of MF was defined as the index event. The follow-up period was five years. The outcomes assessed were survival, transformation to AML (C92.0 or increase in blasts over 20%, as defined by the World Health Organization [1]), cachexia (R64), systemic inflammatory response syndrome (SIRS), (R65), venous thrombosis (I80, I81, I82) and hemorrhage (R04, R58, K62.5). Patients with corresponding outcomes before the index event were excluded. These events were selected as AML transformation, hemorrhage, thrombosis and infections are the most frequent causes of death of myelofibrosis patients [29,30], while cachexia is an established criterion of disease progression [31]. Arterial thrombotic events were not assessed as outcomes in the present study as their evaluation would have required baseline adjustment of the study cohorts regarding competing cardiovascular risk factors and their management, which cannot be provided by current TriNetX analysis tools.

To evaluate the validity of the IPSS, we established a simplified IPSS scoring system for criteria available through TriNetX. For each fulfilled criterion (age above 65 years, hemoglobin < 10 g/dL, and leukocyte count > 25 × 10^3^/µL at diagnosis), one point is assigned, making 0 points the lowest and 3 points the highest possible score. No propensity score matching was performed for the simplified IPSS comparison to assess its validity in clinical practice without considering further parameters.

Data accessible via TriNetX are presented in an aggregated form and only contains anonymized data as per the de-identification standard defined by the US Health Insurance Portability and Accountability Act (HIPAA) in section §164,514(a). As this study exclusively used de-identified anonymized EMRs, it did not require Institutional Review Board approval or written informed consent. The study is in accordance with the STROBE guidelines and the Declaration of Helsinki.

### 2.3. Statistical Analysis

All statistical analyses were performed on the TriNetX analytics platform which offers statistical tools to analyze the aggregated patient data; the user of the platform cannot access individual patient data for reasons of data protection and confidentiality. Differences in survival were assessed through Kaplan–Meier analysis with the Log-Rank test. Risk ratios (RR) with their corresponding 95% confidence intervals (CI 95%) were calculated for the different cohorts. The calculated risk ratios are defined as relative risk for the respective event for the respective cohort 1 compared to respective cohort 2. The threshold of statistical significance was set at *p* ≤ 0.05.

## 3. Results

In total, the network included 64,300 patients with myelofibrosis on 16 March 2024. The course of disease with either known death within 5 years or visit after 5 years was documented for 37,513 patients from 77 HCOs. Of these patients, 638 (1.7%) had a known history of previous ET, and 455 (1.2%) had a history of PV. The characteristics of the study cohort are shown in Table 2.

### 3.1. Independent Impact of Parameters

#### 3.1.1. Impact of Age

**Figure 1:**
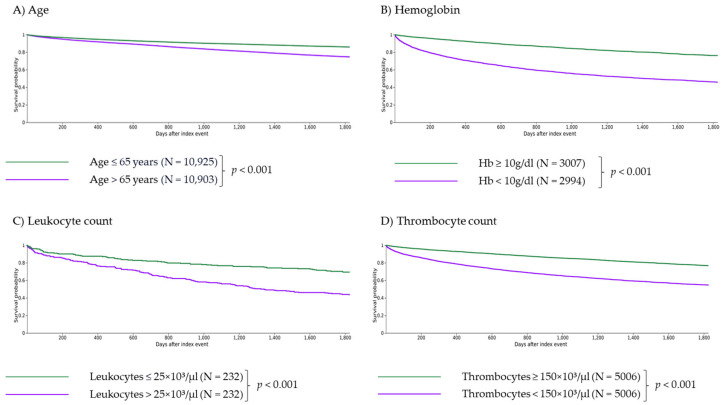

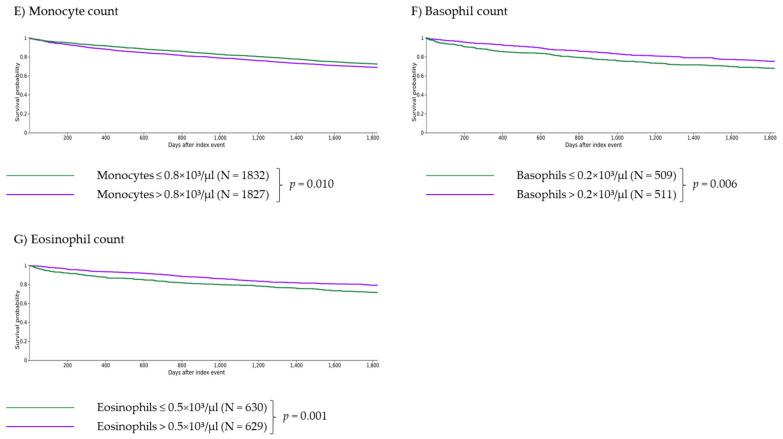
Independent impact of age and laboratory parameters on 5-year survival of myelofibrosis patients. Age > 65 years (**A**), anemia with Hb < 10 g/dL (**B**), severe leukocytosis with leukocytes > 25 × 10^3^/µL (**C**), thrombocytopenia with thrombocytes <150 × 10^3^/µL (**D**) and monocytosis with monocytes > 0.8 × 10^3^/µL (**E**) are independent criteria for survival (with *p* < 0.001 for (**A**–**D**) and *p* = 0.010 for (**E**)). Patients with basophils > 0.2 × 10^3^/µL or eosinophils > 0.5 × 10^3^/µL showed significantly longer survival than patients with basophils ≤ 0.2 × 10^3^/µL or eosinophils ≤ 0.5 × 10^3^/µL ((**F**,**G**), *p* = 0.006 and *p* = 0.001, respectively).

Age > 65 years was associated with significantly higher risk of death (RR 1.798, CI 95% 1.700–1.903, Table 3; survival 74.63% vs. 85.90% at five years, *p* < 0.001, Figure 1A), AML transformation (RR 1.548, CI 95% 1.251–1.915, Table 3), hemorrhage (RR 1.280, CI 95% 1.171–1.400, Table 3) and thrombosis (RR 1.305, CI 95% 1.166–1.461, Table 3), yet showed no significant impact on cachexia (RR 1.189, CI 95% 0.967–1.461, Table 3) or SIRS (RR 1.093, CI 95% 0.974–1.226, Table 3). Patients over the age of 65 years significantly less frequently received an allogeneic stem cell transplantation than patients aged 65 years or younger (RR 0.274, CI 95% 0.182–0.412; data not shown).

#### 3.1.2. Impact of Anemia

Anemia (Hb < 10 g/dL) was associated with significantly lower survival (RR 2.278, CI 95% 2.120–2.448, Table 3; survival 43.79% vs. 74.11% at five years, *p* < 0.001, Figure 1B) as well as a significantly higher risk of AML transformation (RR 6.096, CI 95% 4.124–9.010, Table 3), cachexia (RR 3.052, CI 95% 2.264–4.114, Table 3), SIRS (RR 2.970, CI 95% 2.485–3.550, Table 3), hemorrhage (RR 1.406, CI 95% 1.206–1.641, Table 3) and thrombosis (RR 1.919, CI 95% 1.587–2.320, Table 3).

#### 3.1.3. Impact of Leukocytosis

Severe leukocytosis (leukocytes > 25 × 10^3^/µL) was associated with significantly higher risk of death (RR 1.845, CI 95% 1.474–2.309, Table 3; survival 43.53% vs. 69.40% at five years, *p* < 0.001, Figure 1C) and AML transformation (RR 3.377, CI 95% 1.702–6.702, Table 3), yet showed no significant impact on cachexia (RR 1.917, CI 95% 0.911–4.032, Table 3), SIRS (RR 1.130, CI 95% 0.625–2.044, Table 3), hemorrhage (RR 1.443, CI 95% 0.882–2.360, Table 3) and thrombosis (RR 1.505, CI 95% 0.808–2.802, Table 3).

#### 3.1.4. Impact of Thrombocytopenia

Patients with thrombocytopenia (platelets < 150 × 10^3^/µL) showed significantly lower survival (RR 2.032, CI 95% 1.910–2.162, Table 3; survival 56.57% vs. 78.67% at five years, *p* < 0.001, Figure 1D) as well as a higher risk for AML transformation (RR 5.632, CI 95% 4.365–7.268, Table 3), cachexia (RR 1.799, CI 95% 1.397–2.315, Table 3), SIRS (RR 2.150, CI 95% 1.858–2.487, Table 3), hemorrhage (RR 1.421, CI 95% 1.257–1.605, Table 3) and thrombosis (RR 1.579, CI 95% 1.357–1.837, Table 3).

#### 3.1.5. Impact of Monocytosis

Monocytosis (monocytes > 0.8 × 10^3^/µL) had an adverse impact on survival (RR 1.126, CI 95% 1.018–1.245; survival 68.91% vs. 72.38% at five years, *p* = 0.010, Figure 1E) and on the risk of SIRS (RR 1.407, CI 95% 1.092–1.811, Table 3) and thrombosis (RR 1.428, CI 95% 1.101–1.853, Table 3), and no significant impact on AML transformation (RR 1.122, CI 95% 0.714–1.763, Table 3), cachexia (RR 0.941, CI 95% 0.583–1.519, Table 3), and hemorrhage (RR 1.046, CI 95% 0.850–1.288, Table 3).

#### 3.1.6. Impact of Basophilia

Patients with basophilia (basophils > 0.2 × 10^3^/µL) showed lower risk of death (RR 0.776, CI 95% 0.637–0.945; survival 75.15% vs. 67.98% at five years, *p* = 0.006; Figure 1F). There was no significant impact on the risk of AML transformation (RR 1.640, CI 95% 0.875–3.074, Table 3), cachexia (RR 1.482, CI 95% 0.672–3.268, Table 3), SIRS (RR 1.022, CI 95% 0.636–1.642, Table 3), hemorrhage (RR 1.165, CI 95% 0.790–1.717, Table 3) and thrombosis (RR 1.106, CI 95% 0.717–1.704, Table 3).

#### 3.1.7. Impact of Eosinophilia

Eosinophilia (eosinophils > 0.5 × 10^3^/µL) was associated with a significantly lower risk of death (RR 0.734, CI 95% 0.604–0.893; survival 79.01% vs. 71.43% at five years, *p* = 0.001, Figure 1G). Eosinophilia had no significant impact on the risk of AML transformation (RR 0.662, CI 95% 0.322–1.363, Table 3), cachexia (RR 0.836, CI 95% 0.364–1.921, Table 3), SIRS (RR 0.920, CI 95% 0.593–1.427, Table 3), hemorrhage (RR 0.966, CI 95% 0.688–1.356, Table 3) and thrombosis (RR 1.161, CI 95% 0.731–1.844, Table 3).

### 3.2. Impact of the Simplified IPSS Score

#### 3.2.1. Comparison of 0 vs. 1 Point

**Figure 2:**
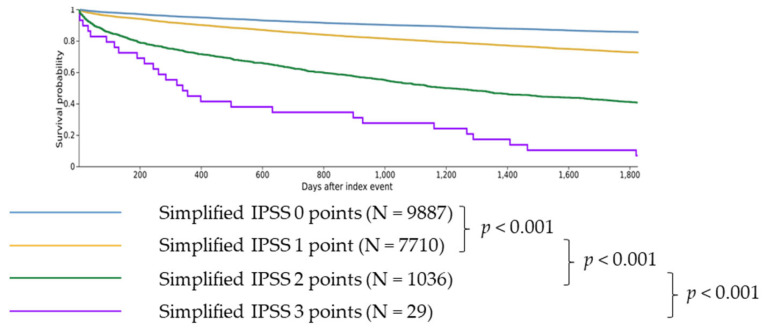
Difference in 5-year survival for patients with simplified IPSS scores from 0 to 3. Patients with 0 simplified IPSS points showed longer survival than patients with 1 simplified IPSS point (*p* < 0.001). Patients with 1 simplified IPSS point showed longer survival than patients with 2 simplified IPSS points (*p* < 0.001). Patients with 2 simplified IPSS points showed longer survival than patients with 3 simplified IPSS points (*p* < 0.001).

Overall, 1 point (vs. 0 points) was associated with significantly higher risk of death (RR 1.932, CI 95% 1.815–2.056, Table 4; survival 73.71% vs. 86.40% at five years, *p* < 0.001, Figure 2) as well as a significantly higher risk of AML transformation (RR 2.515, CI 95% 1.934–3.271, Table 4), cachexia (RR 1.837, CI 95% 1.470–2.296, Table 4), SIRS (RR 1.609, CI 95% 1.429–1.812, Table 4), hemorrhage (RR 1.370, CI 95% 1.242–1.512, Table 4) and thrombosis (RR 1.429, CI 95% 1.265–1.614, Table 4).

#### 3.2.2. Comparison of 1 vs. 2 Points

Patients with 2 points (vs. 1 point) showed significantly higher risk of death (RR 2.255, CI 95% 2.118–2.402, Table 4; survival 40.67% vs. 73.71% at five years, *p* < 0.001, Figure 2) as well as a higher risk of AML transformation (RR 4.609, CI 95% 3.616–5.875, Table 4), cachexia (RR 2.165, CI 95% 1.607–2.918, Table 4), SIRS (RR 1.858, CI 95% 1.560–2.213, Table 4), hemorrhage (RR 1.287, CI 95% 1.077–1.537, Table 4), and thrombosis (RR 1.497, CI 95% 1.221–1.836, Table 4).

#### 3.2.3. Comparison of 2 vs. 3 Points

Patients with 3 points (vs. 2 points) showed significantly higher risk of death (RR 1.571, CI 95% 1.406–1.756, Table 4; survival 6.90% vs. 40.67% at five years, *p* < 0.001, Figure 2) as well as a higher risk of AML transformation (RR 3.623, CI 95% 2.129–6.165, Table 4), cachexia (RR 6.604, CI 95% 3.749–11.631, Table 4), SIRS (RR 2.489, CI 95% 1.479–4.191, Table 4) and hemorrhage (RR 3.015, CI 95% 1.827–4.976, Table 4) and thrombosis (RR 3.312, CI 95% 1.948–5.630, Table 4).

## 4. Discussion

Our study confirms the effect of the established risk factors of age, leukocytosis, thrombocytopenia and anemia on the survival probability of patients with MF and associated complications, including transformation to AML, cachexia, SIRS, thrombosis and hemorrhage. Of note, monocytosis (>0.8 × 10^3^/µL) was associated with inferior survival, whereas basophilia and eosinophilia were associated with improved survival.

Anemia had a significant impact on both survival and the likelihood of MF-associated complications. Advanced age solely affected survival and, to a lesser degree, the likelihood for progression to AML as well as hemorrhage and thrombosis, but not the other examined secondary events, suggesting that reduced life expectancy can at least partially be attributed to the physiological aging process and, in addition, might be related to the limited treatment options available for older individuals [32], which is reflected in this study by a significantly lower rate of allogeneic stem cell transplantations compared to younger individuals. Leukocytosis significantly affected survival and AML transformation, not the other assessed secondary events, which could be attributable to the relatively low number of patients included after propensity score matching (232 patients for each of the two cohorts).

The original IPSS did not include thrombocytopenia as it did not show an additional impact independent of the presence of anemia [8]. Our study demonstrated that thrombocytopenia, independent of the presence of anemia, has a significant and clinically relevant impact on both survival and the likelihood of occurrence of the investigated secondary events. Thrombocytopenia was included as a criterion in newer generations of the IPSS, such as DIPSS and DIPSS Plus [9,10], MIPSS70+ [13], as well as for secondary MF, MYSEC-PM [12]. While a co-occurrence of anemia and thrombocytopenia can be interpreted as a sign of more advanced disease and bone marrow failure [33], it is possible that thrombocytopenia might also be linked to an inflammation-induced upregulation of the coagulation cascade due to the high circulating levels of proinflammatory cytokines typically seen in MF patients [31,34], which would be indicative of increased disease activity independent of progressive bone marrow failure.

The current research on the risk of thrombosis in myelofibrosis presents a heterogeneous picture. Previous studies indicate an increased thrombosis risk for myelofibrosis patients [30,35], and the international prognostic score for thrombosis in essential thrombocythemia (IPSET) has been validated for prefibrotic myelofibrosis [36]. On the other hand, it has been reported that a low IPSS and the presence of a JAK2 V617F mutation are correlated with a higher risk of thrombosis [37]. The data from the present study suggest that the risk of thrombosis increases with IPSS scores. Interestingly, both hemorrhage and thrombosis risks were higher for patients with thrombocytopenia, further suggesting an increase in platelet consumption due to disease-induced hemostatic dysregulation as a pathophysiological mechanism [30,38]. Further research is necessary to assess the pathophysiological mechanism explaining this divergence and to assess the drivers of thrombosis risk in myelofibrosis in order to identify patients potentially benefiting from antithrombotic prophylaxis.

The pathophysiology of MF as disease group characterized by clonal myeloid expansion suggests that an expansion of monocytes, basophils and eosinophils might indicate increased disease activity. Monocytosis has previously been linked to a poor prognosis in PMF patients [39]; the effect was less pronounced in the present study. Eosinophilia is commonly seen in patients with MPN and is interpreted as sign of a perturbed hematopoiesis [40]. Basophilia was previously linked to an accelerated phase of the course of PMF with an increased risk of AML transformation and has been linked to an inferior overall survival in patients with PMF [41,42]. Basophils plus eosinophils over 15% are considered an unfavorable sign for chronic myeloid leukemia (CML) in the SOKAL score [21], while the EURO scores includes both high eosinophils and basophils as independent risk factors for an adverse prognosis [22]. Interestingly, in the present study, both eosinophilia and basophilia seemed to have a favorable effect on survival. This relationship could be caused by confounders not yet identified. However, we speculate that this finding could be attributable to myelofibrosis subtypes, as MF patients with high JAK2 V617F allele burden display increased basophile counts [43] and also improved survival, which might indicate patients with post-PV myelofibrosis that evolved from an undiagnosed PV [44]. Further research is required to confirm the possible link between basophilia and a more favorable outcome in myelofibrosis, and to assess whether eosinophilia and basophilia indicate distinct subtypes of MF.

There are limitations of the present study linked to constraints regarding the availability of data and analysis tools through TriNetX. One limitation is the difficulty to discriminate between primary and secondary myelofibrosis. Only approximately 3% of MF patients in this study had a history of either PV or ET. Considering that approximately 10% of patients with ET and PV progress to MF [45] and the comparable incidence of the classical MPNs [14,15,16], this suggests potential incomplete assessment of the patients’ history in the EMR of the respective HCO and inaccuracies of discrimination between primary and secondary MF. Moreover, in the present study, a transformation to AML could only be observed in 1.3% of cases, while the literature suggests five-year transformation rates of 10% for PMF [2], suggesting insufficient documentation of the patient history. Nevertheless, provided there is an absence of systemic bias in capturing patient histories across diverse cohorts, the deficiencies in documenting disease progression become inconsequential in the assessment of relative risk. Regarding survival data, the patient cohort was selected so that only patients for whom survival or death were known after 5 years were included. The five-year survival rate of c. 78% therefore fell within the range suggested by the literature [2,46]. Furthermore, certain outcomes cannot be clearly delineated in nature. While cachexia is expectedly attributable to the investigated disease myelofibrosis in most cases, systemic inflammatory response syndrome can be directly caused by the disease or indirectly through a severe infection resulting from immunodeficiency [47]. The ICD code I80, which is part of the thrombosis definition in the present paper, also includes phlebitis; likewise, the outcome cachexia is not unambiguously defined, leading to potentially diverging assessment in the different HCOs. These limitations arise inevitably from the nature of a study based on aggregated EMRs, as they do not allow the user to conduct analyses beyond specific predefined categories.

Not all criteria of the IPSS can be validated using TriNetX. Therefore, an expansion of the database or large population studies with alternative data collection methods are needed. Dynamic models require further statistical models capable of assessing the impact of time-dependent covariates which are, as of March 2024, not generally available on TriNetX. Assessment of mutation-related criteria included in the MIPSS70 requires an expansion of the availability of genetic information on TriNetX, which is relatively limited at the time of the publication of this study.

As a proof of concept, our study also included a simplified IPSS that only encompassed the criteria of the original IPSS reliably available through TriNetX (age, hemoglobin, and leukocytes at the time of diagnosis). It was demonstrated that patients with higher scores had an inferior prognosis on survival and the occurrence of MF-associated events and comorbidities, which highlights the utility of EMRs to conceive and validate prognostic scores. Moreover, the simplified IPSS allows for the stratification of patient risk without relying on blast count and constitutional symptoms, which lack inter-individual reproducibility and are sensitive to subjective perception of healthcare staff or patient [48,49,50].

In conclusion, this study demonstrates that EMR datasets provide an immense power to identify prognostic factors and to establish and validate prognostic scores. Our analysis provides empirical evidence that age, leukocytosis, anemia and thrombocytopenia constitute independent prognostic factors in MF patients. Additionally, our findings suggest the inclusion of monocytosis as potential negative prognostic factor, and eosinophilia and basophilia as potential positive prognostic indicators in MF. To fully understand the interplay between these factors and further, mutation-based factors, as well as their underlying pathophysiological mechanisms, further research is required. Moreover, it is important to investigate whether the impact of these factors varies among different patient populations. This research is crucial for achieving an optimal prognosis for each individual patient and aiding in informed treatment decisions.

## 5. Conclusions

The present study confirmed the impact of established risk factors like high age, anemia, thrombocytopenia and leukocytosis on survival and complications, and novel prognostic factors like monocytosis, eosinophilia and basophilia could be identified. Thus, the present study confirmed the utility of TriNetX EMRs to determine risk factors and to establish and to validate clinical scores for rare diseases like myelofibrosis.

## Figures and Tables

**Table 1:** Definition of assessed cohorts.

	Cohort 1	Cohort 2
Age	>65 years	≤65 years
Hemoglobin (Hb)	<10 g/dL	≥10 g/dL
Leukocytes	>25 × 10^3^/µL	≤25 × 10^3^/µL
Platelets	<150 × 10^3^/µL	≥150 × 10^3^/µL
Monocytes	>0.8 × 10^3^/µL	≤0.8 × 10^3^/µL
Basophiles	>0.2 × 10^3^/µL	≤0.2 × 10^3^/µL
Eosinophiles	>0.5 × 10^3^/µL	≤0.5 × 10^3^/µL

**Table 2:** Characteristics of the study cohort.

Attribute	
**Total cohort**, *n* (%)	37,513 (100%)
**Sex**, *n* (%)	
Female	19,976 (53.3%)
Male	15,394 (41.0%)
Unkown	2143 (5.7%)
**Age at diagnosis**	
Mean ± SD	60.3 + 17.5
**Race**	
White	25,963 (69.2%)
Unknown	6806 (18.1%)
Black or African American	2907 (7.7%)
Asian	802 (2.1%)
Other	1035 (2.8%)
**Laboratory** (mean ± SD)	
Hemoglobin (in g/dL) in Blood	13.1 ± 2.0
Leukocytes (in ×10^3^/µL) in Blood	12.3 ± 146
Platelets (in ×10^3^/µL) in Blood	230 ± 100
Monocytes (in ×10^3^/µL) in Blood	8.4 ± 3.6
Basophiles (in ×10^3^/µL) in Blood	0.6 ± 0.8
Eosinophiles (in ×10^3^/µL) in Blood	2.5 ± 2.5
**Outcome**	
Five-year survival rate (in %)	78.4%
Documented five-year AML progression (in %)	1.3%
Documented five-year cachexia rate (in %)	1.6%
Documented five-year SIRS rate (in %)	4.9%
Documented five-year hemorrhage rate (in %)	8.0%
Documented five-year thrombosis rate (in %)	5.2%

**Table 3:** Risk Ratio (RR) and 95% Confidence Interval (95% CI) for impact of independent parameters (rows) on events (columns) on events for myelofibrosis patients within 5 years post-diagnosis.

	Death		AML Transformation		Cachexia		SIRS		Hemorrhage		Thrombosis	
Risk Factor	RR	(95% CI)	RR	(95% CI)	RR	(95% CI)	RR	(95% CI)	RR	(95% CI)	RR	(95% CI)
Age	1.798	(1.700, 1.903)	1.548	(1.251, 1.915)	1.189	(0.967, 1.461)	1.093	(0.974, 1.226)	1.280	(1.171, 1.400)	1.305	(1.166, 1.461)
Anemia	2.278	(2.120, 2.448)	6.096	(4.124, 9.010)	3.052	(2.264, 4.114)	2.970	(2.485, 3.550)	1.406	(1.206, 1.641)	1.919	(1.587, 2.320)
Leuko-cytosis	1.845	(1.474, 2.309)	3.377	(1.702, 6.702)	1.917	(0.911, 4.032)	1.130	(0.625, 2.044)	1.443	(0.882, 2.360)	1.505	(0.808, 2.802)
Thrombo-cytopenia	2.032	(1.910, 2.162)	5.632	(4.365, 7.268)	1.799	(1.397, 2.315)	2.150	(1.858, 2.487)	1.421	(1.257, 1.605)	1.579	(1.357, 1.837)
Monocytosis	1.126	(1.018, 1.245)	1.122	(0.714, 1.763)	0.941	(0.583, 1.519)	1.407	(1.092, 1.811)	1.046	(0.850, 1.288)	1.428	(1.101, 1.853)
Basophilia	0.776	(0.637, 0.945)	1.640	(0.875, 3.074)	1.482	(0.672, 3.268)	1.022	(0.636, 1.642)	1.165	(0.790, 1.717)	1.106	(0.717, 1.704)
Eosinophilia	0.734	(0.604, 0.893)	0.662	(0.322, 1.363)	0.836	(0.364, 1.921)	0.920	(0.593, 1.427)	0.966	(0.688, 1.356)	1.161	(0.731, 1.844)

**Table 4:** Risk Ratio (RR) and 95% Confidence Interval (95% CI) for impact of the simplified IPSS score on events for myelofibrosis patients within 5 years post-diagnosis.

	Death		AML Transformation		Cachexia		SIRS		Hemorrhage		Thrombosis	
Simplified IPSS	RR	(95% CI)	RR	(95% CI)	RR	(95% CI)	RR	(95% CI)	RR	(95% CI)	RR	(95% CI)
0 vs. 1	1.932	(1.815, 2.056)	2.515	(1.934, 3.271)	1.837	(1.470, 2.296)	1.609	(1.429, 1.812)	1.370	(1.242, 1.512)	1.429	(1.265, 1.614)
1 vs. 2	2.255	(2.118, 2.402)	4.609	(3.616, 5.875)	2.165	(1.607, 2.918)	1.858	(1.560, 2.213)	1.287	(1.077, 1.537)	1.497	(1.221, 1.836)
2 vs. 3	1.571	(1.406, 1.756)	3.623	(2.129, 6.165)	6.604	(3.749, 11.631)	2.489	(1.479, 4.191)	3.015	(1.827, 4.976)	3.312	(1.948, 5.630)

## Data Availability

The data was pulled from the constantly updated platform TriNetX and represents the status as of 16 March 2024. Access: https://live.trinetx.com/.

## Supplementary Materials

The following supporting information can be downloaded at: https://www.mdpi.com/article/10.3390/cancers16071416/s1, Table S1: Patient characteristics.
