# Supplementary material for: Real-World Electronic Medical Records Data Identify Risk Factors for Myelofibrosis and Can Be Used to Validate Established Prognostic Scores
Source: Cancers (Basel). 2024 Apr 5;16(7):1416. doi: 10.3390/cancers16071416 (PMC11011132; doi:10.3390/cancers16071416)
Supplement: Supplementary file 1 [file cancers-16-01416-s001.zip › cancers-2921716-supplementary.pdf]

Supplementary Table S1: Patient characteristics

A) Age

Cohort 1 (Age > 65 years, N = 10,975) and cohort 2 (Age ≤ 65 years, N = 10,975) characteristics after propensity score matching. Cohort sizes may differ from the numbers indicated in Figure 1 due to the exclusion of patients with outcome prior to the index event.

Cohort	Parameter	Mean ± SD	Patients	% of Cohort
1	Age at Diagnosis	71.9 +/- 4.5		
2		49.3 +/- 13.3		
1	Female		5149	46.9%
2			5137	46.8%
1	Male		4967	45.3%
2			4979	45.4%
1	Black or African American		574	5.2%
2			577	5.3%
1	White		7934	72.3%
2			7935	72.3%
1	Asian		215	2.0%
2			210	1.9%
1	Other Race		209	1.9%
2			206	1.9%
1	Hemoglobin (in g/dl) in Blood	12.7 +/- 2.3		
2		13.0 +/- 2.4		
1	Platelets (in $\times 10^3/\mu l$) in Blood	216.8 +/- 110.2		
2		228.0 +/- 123.5		
1	Monocytes (in $\times 10^3/\mu l$) in Blood	2.5 +/- 29.3		
2		4.2 +/- 46.6		
1	Basophils (in $\times 10^3/\mu l$) in Blood	0.2 +/- 2.5		
2		0.2 +/- 2.7		
1	Eosinophils (in $\times 10^3/\mu l$) in Blood	0.3 +/- 3.4		
2		0.3 +/- 3.7		
1	Leukocytes (in $\times 10^3/\mu l$) in Blood	7.6 +/- 9.2		
2		14.2 +/- 170.1		

Supplementary Table S1: Patient characteristics

B) Anemia

Cohort 1 (Hb < 10mg/dl, N = 3021) and cohort 2 (Hb ≥ 10mg/dl, N = 3021) characteristics after propensity score matching. Cohort sizes may differ from the numbers indicated in Figure 1 due to the exclusion of patients with outcome prior to the index event.

Cohort	Parameter	Mean ± SD	Patients	% of Cohort
1	Age at Diagnosis	61.5 +/- 18.8		
2		62.2 +/- 17.7		
1	Female		1560	51.6%
2			1596	52.8%
1	Male		1386	45.9%
2			1361	45.1%
1	Black or African American		394	13.0%
2			399	13.2%
1	White		1735	57.4%
2			1719	56.9%
1	Asian		67	2.2%
2			79	2.6%
1	Other Race		84	2.8%
2			77	2.5%
1	Hemoglobin (in g/dl) in Blood	8.7 +/- 1.3		
2		13.3 +/- 1.6		
1	Platelets (in $\times 10^3/\mu l$) in Blood	196.7 +/- 160.7		
2		216.8 +/- 107.7		
1	Monocytes (in $\times 10^3/\mu l$) in Blood	3.2 +/- 40.0		
2		12.6 +/- 79.6		
1	Basophils (in $\times 10^3/\mu l$) in Blood	0.1 +/- 1.3		
2		0.8 +/- 5.2		
1	Eosinophils (in $\times 10^3/\mu l$) in Blood	0.3 +/- 3.4		
2		0.8 +/- 6.5		
1	Leukocytes (in $\times 10^3/\mu l$) in Blood	9.8 +/- 60.9		
2		16.7 +/- 195.5		

Supplementary Table S1: Patient characteristics

C) Leukocytosis

Cohort 1 (Leukocytes > 25×10³/µl, N = 233) and cohort 2 (Leukocytes ≤ 25×10³/µl, N = 233) characteristics after propensity score matching. Cohort sizes may differ from the numbers indicated in Figure 1 due to the exclusion of patients with outcome prior to the index event.

Cohort	Parameter	Mean ± SD	Patients	% of Cohort
1	Age at Diagnosis	65.2 +/- 15.8		
2		65.7 +/- 14.2		
1	Female		95	40.8%
2			97	41.6%
1	Male		131	56.2%
2			126	54.1%
1	Black or African American		12	5.2%
2			10	4.3%
1	White		162	69.5%
2			137	58.8%
1	Asian		10	4.3%
2			10	4.3%
1	Other Race		10	4.3%
2			10	4.3%
1	Hemoglobin (in g/dl) in Blood	11.5 +/- 2.7		
2		12.8 +/- 2.7		
1	Platelets (in ×10³/µl) in Blood	231.5 +/- 181.6		
2		217.4 +/- 116.3		
1	Monocytes (in ×10³/µl) in Blood	98.2 +/- 328.3		
2		127.9 +/- 240.0		
1	Basophils (in ×10³/µl) in Blood	8.3 +/- 15.6		
2		7.5 +/- 15.4		
1	Eosinophils (in ×10³/µl) in Blood	6.3 +/- 20.0		
2		6.5 +/- 20.1		
1	Leukocytes (in ×10³/µl) in Blood	360 +/- 1,061		
2		7.02 +/- 3.19		

Supplementary Table S1: Patient characteristics

D) Thrombocytopenia

Cohort 1 (Thrombocytes < 150×10³/µl, N = 5006) and cohort 2 (Thrombocytes ≥ 150×10³/µl, N = 5006) characteristics after propensity score matching. Cohort sizes may differ from the numbers indicated in Figure 1 due to the exclusion of patients with outcome prior to the index event.

Cohort	Parameter	Mean ± SD	Patients	% of Cohort
1	Age at Diagnosis	62.5 +/- 17.0		
2		62.9 +/- 16.8		
1	Female		1994	39.8%
2			1971	39.4%
1	Male		2845	56.8%
2			2872	57.4%
1	Black or African American		369	7.4%
2			368	7.4%
1	White		111	2.2%
2			102	2.0%
1	Asian		111	2.2%
2			102	2.0%
1	Other Race		148	3.0%
2			153	3.1%
1	Hemoglobin (in g/dl) in Blood	11.7 +/- 2.7		
2		13.1 +/- 2.3		
1	Platelets (in ×10³/µl) in Blood	107.8 +/- 44.0		
2		258.8 +/- 107.6		
1	Monocytes (in ×10³/µl) in Blood	5.2 +/- 49.4		
2		8.0 +/- 67.2		
1	Basophils (in ×10³/µl) in Blood	0.3 +/- 2.7		
2		0.5 +/- 3.8		
1	Eosinophils (in ×10³/µl) in Blood	0.3 +/- 3.3		
2		0.6 +/- 5.9		
1	Leukocytes (in ×10³/µl) in Blood	12.3 +/- 144.7		
2		13.4 +/- 152.5		

Supplementary Table S1: Patient characteristics

E) Monocytosis

Cohort 1 (Monocytes > 0.8×10³/µl, N = 1838) and cohort 2 (Monocytes ≤ 0.8×10³/µl, N = 1838) characteristics after propensity score matching. Cohort sizes may differ from the numbers indicated in Figure 1 due to the exclusion of patients with outcome prior to the index event

Cohort	Parameter	Mean ± SD	Patients	% of Cohort
1	Age at Diagnosis	62.8 +/- 17.9		
2		64.5 +/- 16.7		
1	Female		761	41.4%
2			741	40.3%
1	Male		924	50.3%
2			941	51.2%
1	Black or African American		102	5.5%
2			87	4.7%
1	White		1248	67.9%
2			1308	71.2%
1	Asian		34	1.8%
2			32	1.7%
1	Other Race		47	2.6%
2			37	2.0%
1	Hemoglobin (in g/dl) in Blood	12.8 +/- 2.3		
2		12.9 +/- 2.3		
1	Platelets (in ×10³/µl) in Blood	252.8 +/- 137.8		
2		223.1 +/- 117.3		
1	Monocytes (in ×10³/µl) in Blood	18.1 +/- 98.5		
2		0.5 +/- 0.2		
1	Basophils (in ×10³/µl) in Blood	0.7 +/- 4.3		
2		0.1 +/- 0.2		
1	Eosinophils (in ×10³/µl) in Blood	1.2 +/- 8.2		
2		0.2 +/- 0.3		
1	Leukocytes (in ×10³/µl) in Blood	20.7 +/- 219.0		
2		7.3 +/- 6.9		

Supplementary Table S1: Patient characteristics

F) Basophilia

Cohort 1 (Monocytes > 0.8×10³/µl, N = 513) and cohort 2 (Monocytes ≤ 0.8×10³/µl, N = 513) characteristics after propensity score matching. Cohort sizes may differ from the numbers indicated in Figure 1 due to the exclusion of patients with outcome prior to the index event				
**Cohort**	**Parameter**	**Mean ± SD**	**Patients**	**% of Cohort**
1<br>2	Age at Diagnosis	60.0 +/- 17.3<br>61.1 +/- 18.0		
1<br>2	Female		258<br>242	50.3%<br>47.2%
1<br>2	Male		223<br>225	43.5%<br>43.9%
1<br>2	Black or African American		36<br>30	7.0%<br>5.8%
1<br>2	White		301<br>293	58.7%<br>57.1%
1<br>2	Asian		18<br>14	3.5%<br>2.7%
1<br>2	Other Race		10<br>10	1.9%<br>1.9%
1<br>2	Hemoglobin (in g/dl) in Blood	12.7 +/- 2.4<br>11.6 +/- 2.9		
1<br>2	Platelets (in ×10³/µl) in Blood	251.4 +/- 167.2<br>216.3 +/- 141.2		
1<br>2	Monocytes (in ×10³/µl) in Blood	227.4 +/- 282.7<br>16.6 +/- 110.9		
1<br>2	Basophils (in ×10³/µl) in Blood	11.3 +/- 16.2<br>0.0 +/- 0.1		
1<br>2	Eosinophils (in ×10³/µl) in Blood	14.1 +/- 27.8<br>0.9 +/- 4.9		
1<br>2	Leukocytes (in ×10³/µl) in Blood	25.9 +/- 259.6<br>12.2 +/- 23.2		

Supplementary Table S1: Patient characteristics

G) Eosinophilia

Cohort 1 (Eosinophils > $0.5\times10^3/\mu l$, N = 632) and cohort 2 (Eosinophils ≤ $0.5\times10^3/\mu l$, N = 632) characteristics after propensity score matching. Cohort sizes may differ from the numbers indicated in Figure 1 due to the exclusion of patients with outcome prior to the index event.

Cohort	Parameter	Mean ± SD	Patients	% of Cohort
1	Age at Diagnosis	59.0 +/- 19.0		
2		60.8 +/- 17.5		
1	Female		313	49.5%
2			295	46.7%
1	Male		273	43.2%
2			287	45.4%
1	Black or African American		43	6.8%
2			39	6.2%
1	White		408	64.6%
2			423	66.9%
1	Asian		15	2.4%
2			10	1.6%
1	Other Race		14	2.2%
2			11	1.7%
1	Hemoglobin (in g/dl) in Blood	13.0 +/- 2.2		
2		12.5 +/- 2.5		
1	Platelets (in $\times10^3/\mu l$) in Blood	274.0 +/- 154.8		
2		225.5 +/- 121.4		
1	Monocytes (in $\times10^3/\mu l$) in Blood	84.7 +/- 202.8		
2		1.7 +/- 16.8		
1	Basophils (in $\times10^3/\mu l$) in Blood	4.2 +/- 11.1		
2		0.3 +/- 3.0		
1	Eosinophils (in $\times10^3/\mu l$) in Blood	5.8 +/- 18.2		
2		0.1 +/- 0.1		
1	Leukocytes (in $\times10^3/\mu l$) in Blood	10.8 +/- 13.8		
2		8.9 +/- 9.1		

Supplementary Table S1: Patient characteristics

H) Simplified IPSS Score

Cohort 1 (N = 10,243, simplified IPSS 0 points), cohort 2 (N = 7840, simplified IPSS 1 point), cohort 3 (N = 1049, simplified IPSS 2 points) and cohort 4 (N= 29, simplified IPSS 3 points) characteristics without propensity score matching. Cohort sizes may differ from the numbers indicated in Figure 2 due to the exclusion of patients with outcome prior to the index event.

Cohort	Parameter	Mean ± SD	Patients	% of Cohort
1	Age at Diagnosis	48.7 +/- 13.4		
2	Age at Diagnosis	66.6 +/- 12.7		
3	Age at Diagnosis	71.6 +/- 6.8		
4	Age at Diagnosis	74.5 +/- 4.2		
1	Female		5549	
2	Female		3788	
3	Female		476	
4	Female		10	
1	Male		3907	
2	Male		3450	
3	Male		522	
4	Male		18	
1	Black or African American		871	
2	Black or African American		597	
3	Black or African American		86	
4	Black or African American		0	
1	White		5504	
2	White		6904	
3	White		677	
4	White		21	
1	Asian		218	
2	Asian		118	
3	Asian		19	
4	Asian		10	
1	Other Race		254	
2	Other Race		127	
3	Other Race		20	
4	Other Race		10	
1	Hemoglobin (in g/dl) in Blood	13.4 +/- 1.9		
2	Hemoglobin (in g/dl) in Blood	12.6 +/- 2.3		
3	Hemoglobin (in g/dl) in Blood	10.1 +/- 1.9		
4	Hemoglobin (in g/dl) in Blood	8.7 +/- 1.3		
1	Platelets (in ×10^3/µl) in Blood	238 +/- 103		
2	Platelets (in ×10^3/µl) in Blood	220 +/- 111		
3	Platelets (in ×10^3/µl) in Blood	207 +/- 148		
4	Platelets (in ×10^3/µl) in Blood	229 +/- 149		
1	Monocytes (in ×10^3/µl) in Blood	8.1 +/- 3.5		
2	Monocytes (in ×10^3/µl) in Blood	8.7 +/- 4.1		
3	Monocytes (in ×10^3/µl) in Blood	8.2 +/- 5.7		
4	Monocytes (in ×10^3/µl) in Blood	4.6 +/- 4.7		
1	Basophils (in ×10^3/µl) in Blood	0.6 +/- 0.6		
2	Basophils (in ×10^3/µl) in Blood	0.6 +/- 0.8		
3	Basophils (in ×10^3/µl) in Blood	0.7 +/- 2.3		
4	Basophils (in ×10^3/µl) in Blood	1.7 +/- 2.3		
1	Eosinophils (in ×10^3/µl) in Blood	2.4 +/- 2.6		
2	Eosinophils (in ×10^3/µl) in Blood	2.6 +/- 2.6		
3	Eosinophils (in ×10^3/µl) in Blood	2.2 +/- 2.4		
4	Eosinophils (in ×10^3/µl) in Blood	1.9 +/- 3.0		
1	Leukocytes (in ×10^3/µl) in Blood	8.0 +/- 6.6		
2	Leukocytes (in ×10^3/µl) in Blood	15.0 +/- 187		
3	Leukocytes (in ×10^3/µl) in Blood	20.9 +/- 186		
4	Leukocytes (in ×10^3/µl) in Blood	158 +/- 479		
